# Carbon nanotubes play an important role in the spatial arrangement of calcium deposits in hydrogels for bone regeneration

**DOI:** 10.1007/s10856-016-5740-3

**Published:** 2016-06-20

**Authors:** Giulia Cancian, Gianluca Tozzi, Amirul Ashraf Bin Hussain, Arianna De Mori, Marta Roldo

**Affiliations:** 1 School of Pharmacy and Biomedical Science, University of Portsmouth, St Michael’s Building, White Swan Road, PO1 2DT Portsmouth, UK; 2 School of Engineering, University of Portsmouth, Anglesea Building, Anglesea Road, PO1 3DJ Portsmouth, UK

## Abstract

Age related bone diseases such as osteoporosis are considered among the main causes of reduced bone mechanical stability and bone fractures. In order to restore both biological and mechanical function of diseased/fractured bones, novel bioactive scaffolds that mimic the bone structure are constantly under development in tissue engineering applications. Among the possible candidates, chitosan-based thermosensitive hydrogel scaffolds represent ideal systems due to their biocompatibility, biodegradability, enhanced antibacterial properties, promotion of osteoblast formation and ease of injection, which makes them suitable for less invasive surgical procedures. As a main drawback, these chitosan systems present poor mechanical performance that could not support load-bearing applications. In order to produce more mechanically-competent biomaterials, the combined addition of hydroxyapatite and carbon nanotubes (CNTs) is proposed in this study. Specifically, the aim of this work is to develop thermosensitive chitosan hydrogels containing stabilised single-walled and multi-walled CNTs, where their effect on the mechanical/physiochemical properties, calcium deposition patterns and ability to provide a platform for the controlled release of protein drugs was investigated. It was found that the addition of CNTs had a significant effect on the sol–gel transition time and significantly increased the resistance to compression for the hydrogels. Moreover, in vitro calcification studies revealed that CNTs played a major role in the spatial arrangements of newly formed calcium deposits in the composite materials studied, suggesting that they may have a role in the way the repair of fragile and/or fractured bones occurs in vivo.

## Introduction

Bone is a dynamic, mineralised and highly vascularised tissue whose major functions are body support, motion and organ protection. Major alterations in its structure, due to injuries or metabolic diseases, can cause long-term pain, infection, inflammation and loss of mobility that can lead to a general decrease in functionality and quality of life [1]. Current therapies for complex bone fractures are mainly based on auto- and allo-bone grafts, or on the use of inert materials such as metals and ceramics that replace bone. These invasive strategies have, however, many serious limitations such as an inadequate revascularisation and mineralisation of the bone grafts, risk of infection, rejection and immunoreaction [2].

Bone tissue engineering (BTE) provides an interdisciplinary approach to promote the physiological regeneration of bone tissues, overcoming the downfalls of current methods. BTE research focuses on developing bioactive scaffolds that, mimicking the bone structure, work as temporary matrices for cell proliferation, extracellular matrix deposition and vascularization of newly formed tissue [3, 4]. In order to reach these targets, an ideal scaffold for BTE applications should possess the following properties: have a porous structure; be biocompatible; have good mechanical properties to support tissue ingrowth; be fully biodegradable; resorb while bone formation occurs, eliminating the need for a revision surgery; not form toxic products; and finally deliver bioactive molecules or drugs in a controlled manner where needed [5].

Among the possible scaffolds for tissue engineering, hydrogels represent ideal systems due to their biocompatibility and biodegradability [6, 7]. These gels can be formulated from different natural polymers such as collagen, chitosan, fibrin, hyaluronic acid and alginate [8, 9]. Chitosan is a linear polysaccharide derived from *N*-deacetylation of chitin, which is commonly found in the exoskeletons of crustaceans and insects [10]; its biocompatibility, antibacterial properties and structure amenable to simple chemical modifications, make chitosan a very interesting polymer for biomedical applications [11]. Chitosan only hydrogels present poor bioactive and mechanical properties; however, we have previously demonstrated that the combined addition of hydroxyapatite (HA) and carbon nanotubes (CNTs) can improve gels properties [12]. HA is a natural, nontoxic and bioactive ceramic, shown to enhance osteoconduction, bone bonding and stiffness in scaffolds. Moreover, HA favours deposition of calcium phosphate, improving the bone-matrix interface strength [13, 14]. CNTs are cylindrical molecules of carbon atoms in sp^2^ conformation with outstanding chemical, electrical, mechanical and thermal properties [15]. They have recently been shown to have a role in the differentiation of osteoblasts suggesting that they might have a double function in scaffolds for BTE as structural reinforcement and osteiniductive materials [16]. However, their application is dependent on their successful dispersion in physiological conditions. In fact, CNTs have a tendency to form large bundles in aqueous media, resulting from Van der Waals interaction and π–π stacking [17–19]. Such bundles may cause slippage between nanotubes, become stress concentrators or initiate cracks under applied forces. Many covalent and non-covalent modifications of CNTs have been proposed in order to increase their water dispersability and hence their biocompatibility. Non-covalent modifications, in particular, are based on the absorption or the wrapping of various functional molecules, such as biomolecules, polymers, surfactants and phospholipids around the CNTs [20]. In a previous study, we have demonstrated the possibility of efficiently stabilising single-walled carbon nanotubes with the amphiphilic and self-assembling chitosan derivative *N*-octyl-*O*-sulfate chitosan (NOSC) [21].

In the present study, we developed thermosensitive chitosan hydrogels containing NOSC stabilised CNTs (SWNTs or MWNTs). Our aim was to develop a more flexible formulation strategy by using non-covalent CNTs stabilisation instead of chemical grafting and studying the effects of this strategy on the mechanical and physicochemical properties of the formulations. Image analysis was used to qualitatively and quantitatively assess the effect of CNTs on salts deposition within the formulated hydrogels. The ability of the gel to provide a platform for the controlled release of protein drugs was also evaluated.

## Materials and methods

### Materials

All salts and solvents, unless otherwise stated, were obtained from Fisher Scientific (UK). Sodium phosphate monobasic monohydrate, low viscosity chitosan from shrimp, glycerol phosphate disodium salt hydrate, tris(hydroxymethyl)amino-methane [(HOCH_2_)_3_CNH_2_], albumin from bovine serum (BSA), albumin from chicken egg white (OVA) and QuantiPro™ Bicinchoninic Acid Assay Kit were obtained from Sigma-Aldrich (UK). l(+)-lactic acid 90 % solution in water was obtained from Acros Organics (USA). Dialysis membranes (size 10, MWCO 12–14 kDa) were obtained from Medicell International Ltd (UK). MWCTs and SWCTs were purchased from Cheap Tubes Inc. (USA). The MWCTs used in the study were 8–15 nm in diameter and 10–50 µm in length. The SWCTs used were 1–4 nm in diameter and 5–30 µm in length. Di-potassium hydrogen orthophosphate anhydrous (K_2_HPO_4_) was acquired from British Drug Houses (UK) and troclosene sodium dehydrate from Guest Medical (UK). Water used in all experiments was purified water. NOSC and chitosan modified hydroxyapatite (HACS) were prepared as described previously [12, 21].

### Formulation of hydrogels

Clear solutions of chitosan were prepared by dissolving chitosan (200 mg) in lactic acid (0.1 M, 7 ml), HACS (86 mg) was then added and dispersed by vigorous mixing and sonication. CNTs loaded formulations [CS-HACS-(NOSC-SWNTs) and CS-HACS-(NOSC-MWNTs)] were prepared by addition of stable CNTs suspensions in NOSC (2 ml) to the HACS containing chitosan solutions. NOSC suspensions of CNTs were obtained by adding CNTs (2.5 mg) to a solution of NOSC (1.25 mg/ml) sonicating for 2 h, followed by overnight rest and further sonication for 1 h. At this point all suspensions were brought to a final volume of 9 ml by addition of lactic acid 0.1 M (if needed) and the thermosenstive crosslinker glycerol phosphate was added (1 ml, 1.12 g/ml) to all samples, which were then stirred for 10 min. Gelation was obtained by incubating the formulations at 37 °C. The gels prepared were labelled CS (chitosan control gel), CS-HACS (chitosan gel containing HACS),CS-HACS-(NOSC-SWNTs) and CS-HACS-(NOSC-MWNTs) (gels containing HACS and SWNTs or MWNTs, respectively). All gels were prepared in triplicate; gelation time was evaluated visually by the inversion method [22].

### Texture analysis of the gels

A Texture Analyzer (XT *plus*, Stable Micro Systems Ltd, UK) was used to determine syringeability of the suspensions before gelation as well as gel compressibility. The methods employed have been described previously by Yasmeen et al. [12]. Briefly, for syringeability the liquid formulations were loaded onto 5 ml plastic syringes fitted with 19 G, 25 mm long needles. The vertically clamped syringe was actioned by the instrument probe (10 mm diameter) that compressed the barrel (1 mm/s) to a distance of 40 mm and the initial glide force, dynamic glide force and maximum force were measured. Compressibility tests were performed by depressing a polycarbonate probe (10 mm dia) into the gels at 1 mm/s up to a depth of 5 mm, six measurements were taken before and after the sol–gel transition took place. Gels were formed and tested in 14 ml glass vials. Force/displacement curves were obtained and used to calculate the gel compressibility (N∙mm). All measurements were performed at room temperature.

### Characterisation of the freeze dried hydrogels

Freeze dried hydrogels were prepared from the suspension described above and then transferred into 24 well-plates (2 ml per well), before incubation at 37 °C. Once formed, the gels were flash-freezed and freeze-dried (Edwards Micro Modulyo RV3, UK). The samples were characterized by FT-IR (Varian Spectrometer 640, UK), DVS (Surface Measurement Systems DVS Advantage, UK) and SEM (JEOL JSM-6060 LV Scanning Electron Microscope, UK). In the DVS analysis the mass change of the gels subjected to a changing water vapour partial pressure at 25 °C was recorded. The partial pressure was increased from 0 to 90 % at incremental steps of 10 %, where the next step was reached either after equilibrium or after a maximum time of 360 min. A full adsorption/desorption cycle was performed and the data collected were used to calculate the adsorption/desorption isotherms.

### In vitro calcification assay

Calcification studies were performed to investigate the ability of the composite hydrogels to induce calcium salts deposition. Simulated body fluid (SBF), prepared according to Kokubo and Takadama [23], was used to mimic the in vivo environment, as it contains ions at very similar concentrations to those of human blood plasma. The freeze dried samples were inserted in a 50 ml tube to which 15 ml of SBF were added. Samples were incubated at 37 °C for 7 or 14 days. The SBF was changed every 4 days. Finally, samples were dried under vacuum at 40 °C overnight. Controls were hydrated in deionised water for 5 h and dried as above. All samples were gold-coated with sputter coater (Polaron e500, Quoram Technologies, UK) and analysed by SEM, coupled with EDS [Silicon Drift Detector (SDD)—X-MaxN, Oxford Instruments, UK] and microCT (XTH225, Nikon Systems, UK). The microCT scanner was set to a voltage of 55 kV and a current of 75 μA. With an isotropic voxel size of 10.6 μm and exposure of 2 s, the image acquisition was performed at a rotational step of 0.23° over 360° for 90 min approximately. The images were then analysed using ImageJ (1.48v, NIH, USA) as described below. To quantify calcium deposition, image thresholding (global) based on the grey scale histogram was carried out to distinguish between the different material densities (different attenuation). Total volume of the sample and total volume of the denser material in the sample (i.e. the deposits of calcium salts) were obtained and the percentage of calcium deposition was calculated for each sample (n = 5). EDS images for calcium (Ca) and phosphorus (P) were also analysed after being overlaid to corresponding SEM images. The total area of overlapping for Ca and P onto the total sample surface area was used to calculate percentage of Ca and P salt deposition (n = 3).

### Release of model drugs

Release studies were performed including bovine serum albumin (BSA, 1 mg/ml) in the hydrogels (5 ml). After the gelation of the samples, prepared in a 15 ml tubes (n = 4), PBS (5 ml) was added. The samples were then incubated at 37 °C and at set time points 2 ml of PBS were sampled and replaced with 2 ml of fresh buffer. Release studies were also performed including ovalbumin (OVA, 1 mg/ml) in the hydrogels. The suspensions were transferred (1 g per sample) into a Flot-A-Lyzer G2 dialysis device (cut off 50 kDa) and PBS (15 ml) was added to them. The samples were subsequently incubated at 37 °C and at set time points the sampled buffer was replaced with fresh buffer. Determination of both BSA and OVA concentrations was carried out with a QuantiPro™ Bicinchoninic Acid Assay Kit (Sigma, UK) and samples prepared according to manufacturer’s instructions, reading the UV absorbance at 570 nm. Unloaded gels were used as a control to eliminate any false response due to chitosan interaction with the assay kit. The two model drugs were tested with slightly different methods due to limitations imposed by their molecular weight.

## Results

### Texture analysis of the thermosentive hydrogels

**Fig. 1:**
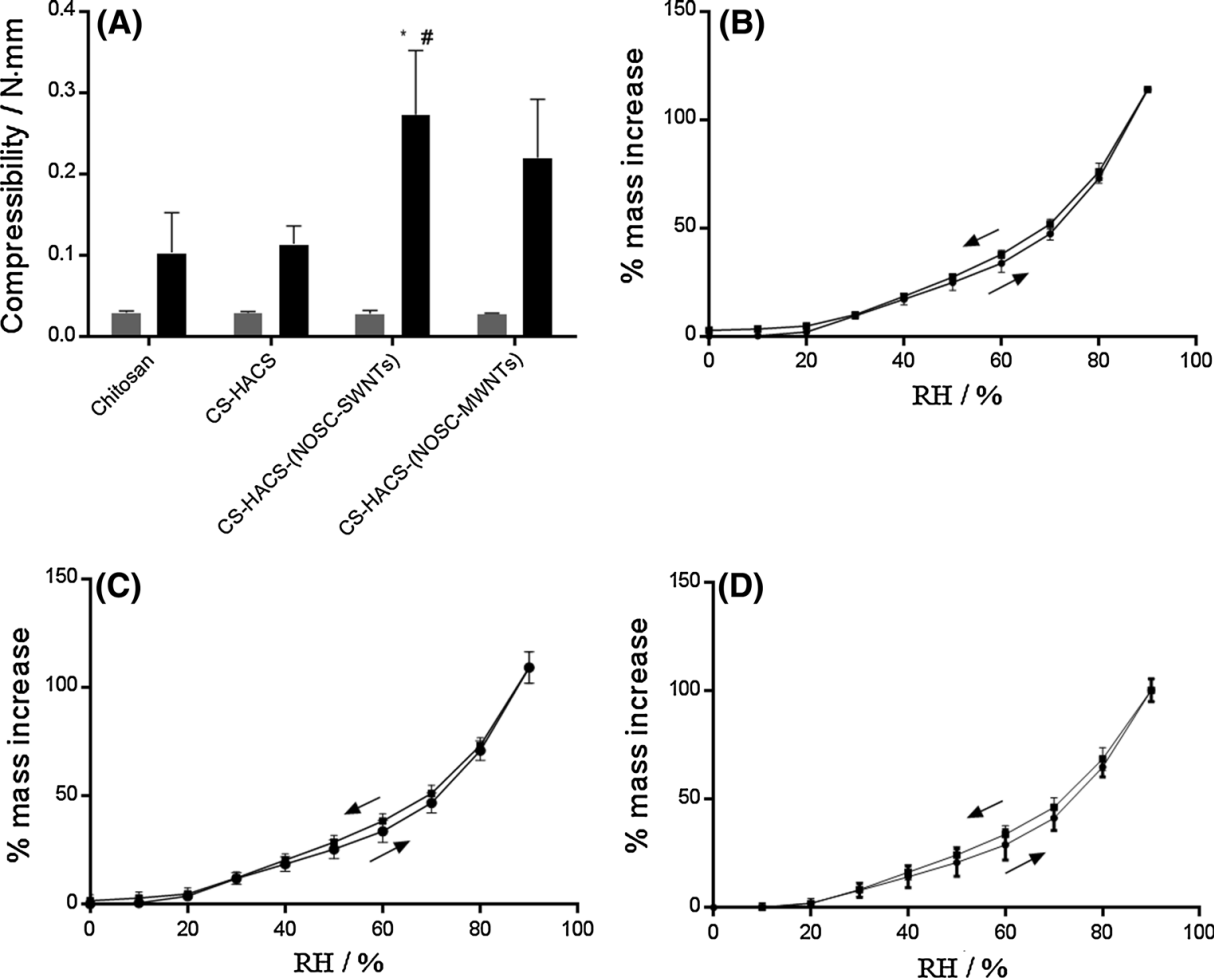
**a** Compressibility of the different formulations before (*grey*) and after (*black*) sol–gel transition. Results are reported as mean ± SD (n = 3). One way ANOVA (α = 0.05) on the values after gelation returned *P* = 0.0131; Tukey’s multiple comparisons test: ^*^ *P* < 0.05, compared to chitosan; ^#^ *P* < 0.05 compared to CS-HACS. Water sorption and desorption isotherms of the freeze dried hydrogels: **b** chitosan; **c** CS-HACS-(NOSC-SWNTs); **d** CS-HACS-(NOSC-MWNTs). Data are presented as mean ± SD (n = 3)

A chitosan only formulation was used as a control to evaluate the effect of the addition of HACS and CNTs suspensions on the physical properties of gels before and after formation. The chitosan only formulation underwent sol–gel transition at 37 °C in 60 min, whereas the addition of HACS and NOSC suspensions of SWNTs and MWNTs reduced this time to 7 and 9 min, respectively. The syringeability of the suspensions was also studied. A force of 30 N was considered as the maximum acceptable force for an injection to be performed without particular patient discomfort [24]; none of the samples tested presented values above 10 N (Table 1) [25]. Furthermore, texture analysis was performed to determine gel compressibility (Fig. 1a).

**Table 1:** Syringeability of composite formulations

	Stiction (N)	Plateau force (N)	End capture (N)
Chitosan	8.36 ± 0.33	8.56 ± 0.29	8.92 ± 0.13
Chitosan-HACS	8.86 ± 0.39	8.56 ± 0.34	9.09 ± 0.34
Chitosan-HACS-(NOSC-SWNTs)	6.69 ± 0.25***	6.40 ± 0.24***	6.56 ± 0.21***
Chitosan-HACS-(NOSC-MWNTs)	7.03 ± 0.15***	6.92 ± 0.15***	7.15 ± 0.22***

Stiction, plateau force and end capture are expressed as mean ± SD (n = 3). One way ANOVA, *P* < 0.001; Tukey–Kramer multiple comparison test

*** *P* < 0.001, compared to the chitosan control

All suspensions tested showed significant difference in strength before and after gelation at 37 °C (at least *P* = 0.0004, two tailed, *t* test with Welch’s correction) apart from the chitosan control. In particular, after gelation the formulation containing SWNTs showed significantly higher values compared to all other formulations (0.273 ± 0.001 N mm).

### Characterisation of the freeze dried hydrogels

**Fig. 2:**
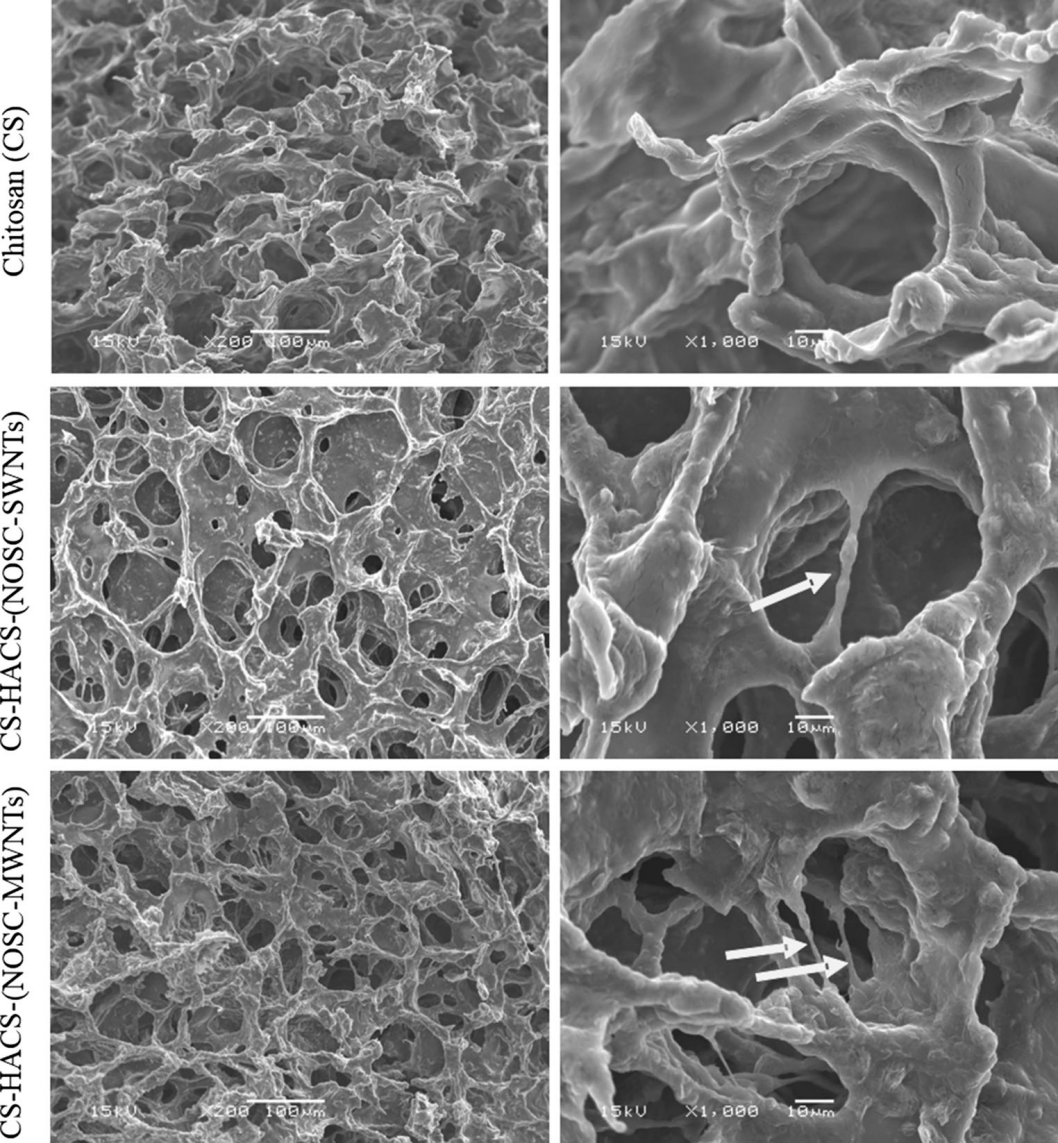
SEM of chitosan, CS-HACS-(NOSC-SWNTs) and CS-HACS-(NOSC-MWNTs) freeze dried hydrogels. *Arrows* indicate coated carbon nanotubes within the hydrogel matrix

The morphology of the freeze-dried hydrogels was evaluated by SEM (Fig. 2). The chitosan only gel appeared to be scaly and rough while the gels containing CNTs showed a more defined porous structure with evidence of coated CNTs filaments bridging the structure; this was previously observed by Ormsby et al. who observed CNTs bridging sub-micron voids and preventing pore coalescence [26].

#### Dynamic vapour sorption

DVS analysis was performed to evaluate the amount and kinetics of water sorption of the hydrogels. Previous work showed that the addition of chitosan grafted CNTs to chitosan hydrogel significantly decreased the hydrophilicity of the formulation [12]; this was not the case when the added CNTs were non-covalently coated with NOSC (Fig. 1b–d). Applying Korsmeyer–Peppas model to the absorption versus time curves, the mechanism of water penetration described by the value of n_p_ > 0.89 was determined to be super case-II transport (Table 2). Hence, it seems that the dynamic swelling behaviour of hydrogels is dependent on the contribution of both the penetrant (water) diffusion and polymer relaxation [27].

**Table 2:** Kinetic parameter values for water penetration in freeze-dried hydrogels

	n_p_	K_p_	R^2^
CS control	1.67 ± 0.15	0.000369 ± 0.000314	0.968 ± 0.015
CS-HACS-(NOSC-SWNTs)	1.79 ± 0.23	0.000260 ± 0.000335	0.973 ± 0.016
CS-HACS-(NOSC-MWNTs)	1.65 ± 0.16	0.000461 ± 0.000298	0.983 ± 0.007

Data are reported as mean ± SD (n = 3)

#### In vitro calcification studies

**Fig. 3:**
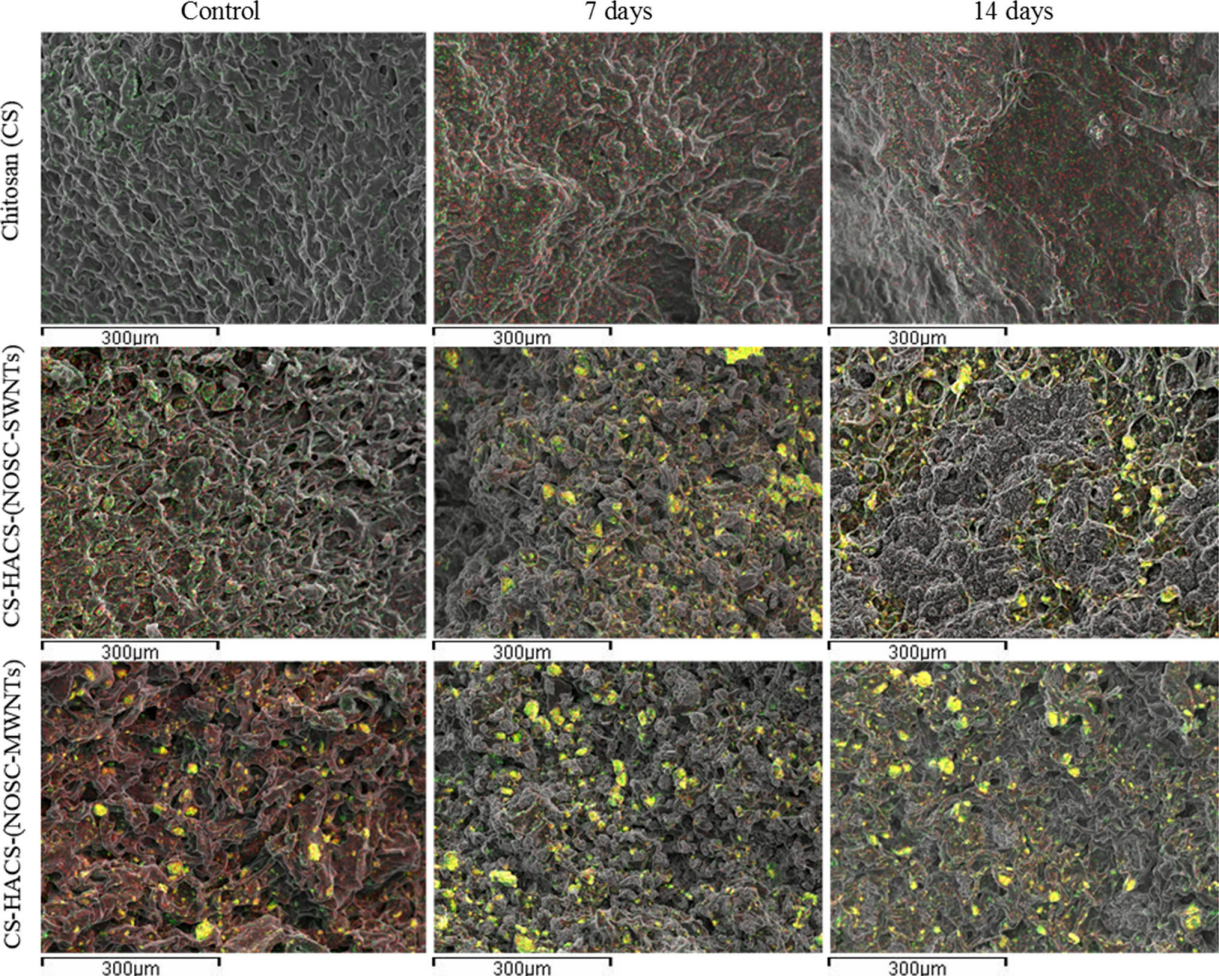
Overlay images of SEM pictures with EDS data: Ca (*green*), P (*red*) and co-localisation of Ca/P (*yellow*) (Color figure online)

**Fig. 4:**
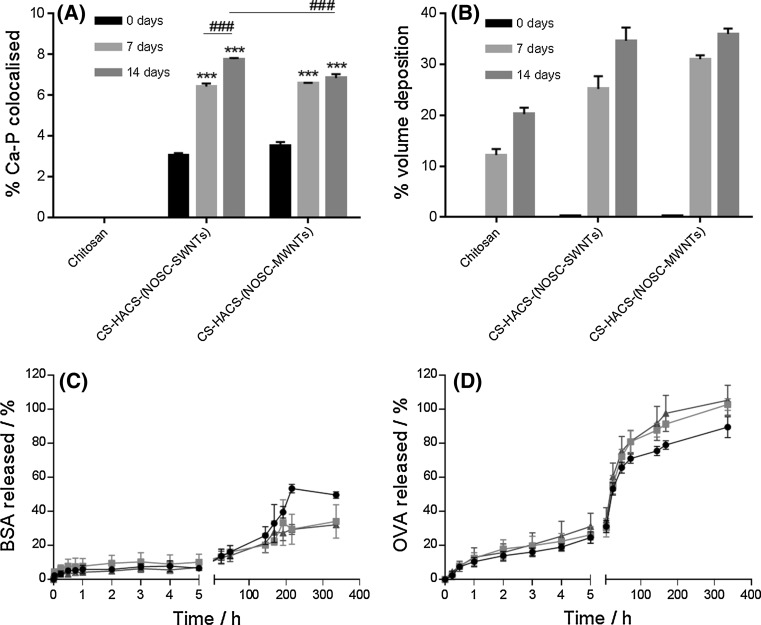
**a** Quantitative analysis of surface calcium deposition presenting % of surface covered by co-localised Ca and P (from SEM). Data are reported as mean ± SD (n = 3). One-way ANOVA (α = 0.05) *P* < 0.0001 for both gels containing CNTs; Tukey’s multicomparison test, ***P < 0.001 compared to control at time zero, ^###^ *P* < 0.001 compared to a specific sample as indicated in the figure. **b** Average % salt deposition on different types of hydrogel samples (from microCT). Data are reported as mean ± SD (n = 5). One-way ANOVA (α = 0.05) *P* < 0.0001 for both gels containing CNTs, Tukey’s multicomparison test, all samples presented *P* < 0.001 compared to control, and *P* < 0.001 was obtained for all comparisons between 7 and 14 days. Release profile of **c** BSA **d** OVA from (*black closed circle*) chitosan; (*red closed square*) CS-HACS-(NOSC-SWNTs); (*blue closed triangle*) CS-HACS-(NOSC-MWNTs) gels. Data are reported as mean ± SD (n = 3) (Color figure online)

Bioactivity of a scaffold proposed for bone regeneration can be evaluated by studying the calcium salts deposition on its surface and internal structure [23, 28]. Samples were soaked in SBF for 7 and 14 days, at 37 °C, and superficial Ca/P co-deposition was evaluated by EDS coupled SEM (Fig. 3); deposition, in the whole volume, of a denser material was also visualised and quantified by microCT. The quantitative analysis of the EDS data (Fig. 4a) revealed that no calcium-phosphate deposits were found in the gels containing only chitosan, both at 7 and 14 days. Samples containing CNTs also contained HACS, this accounted for 3.06 ± 0.09 and 3.52 ± 0.18 % co-localised Ca/P present at time zero, for gels containing SWNTs and MWNTs respectively. Furthermore, the SEM images (Fig. 3) showed that HACS was more homogeneously distributed in the samples containing SWNTs. In both samples an increase in the concentration of co-localised Ca and P was observed, with significantly higher values for the gel containing SWNTs (7.8 ± 0.1 %). Further analysis of areas characterised by Ca/P co-localisation revealed that the Ca/P ratio of the newly formed deposits was 1.55 (Table 3) for gels containing MWNTs, this corresponds to calcium deficient HA. Gels containing SWNTs presented deposits with Ca/P ratio <1.50, suggesting a combination of crystal phases of lower Ca/P values such as monetite, brushite and octacalcium phosphate.

**Table 3:** Ca/P ratio of salt deposits on hydrogels incubated with SBF for 7 and 14 days, *t*-test (paired, two-tailed)

	7 days	14 days
CS-HACS-(NOSC-SWNTs)	1.45 ± 0.54	1.32 ± 0.04
CS-HACS-(NOSC-MWNTs)	1.55 ± 0.30	1.55 ± 0.15*

* *P* < 0.05, compared to the HACS included in the gels which had a Ca/P ratio of 1.34 ± 0.06

**Fig. 5:**
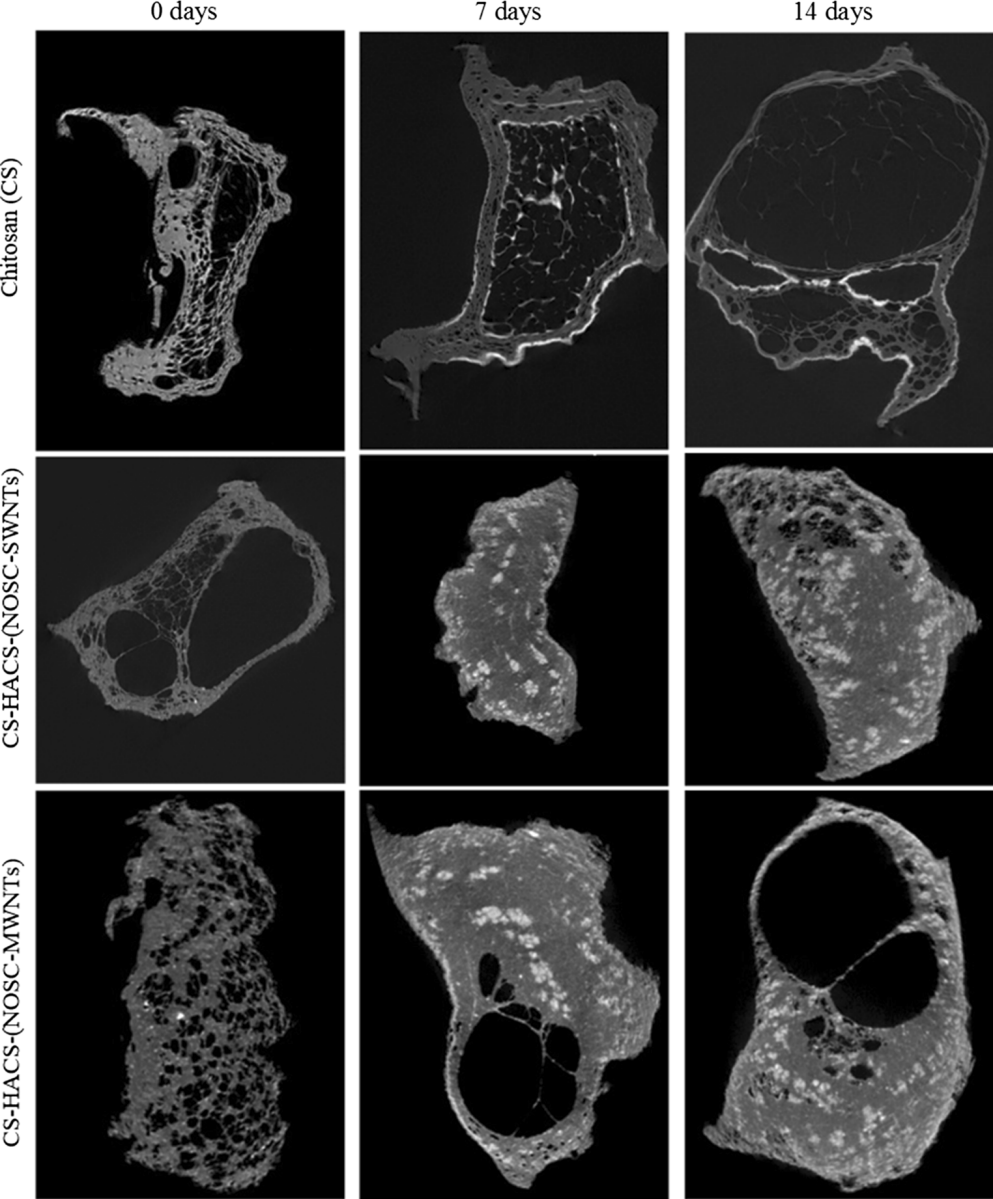
Representative micro CT slices of different gel samples incubated in SBF for 0, 7 and 14 days

Salts deposition was also observed by micro CT (Fig. 4b), were volumes of denser material were identified and quantified, these may represent the deposition of other salts as well as calcium phosphate salts that possess similar x-ray attenuations. The micro CT analysis evidenced differences in the microarchitecture of the hydrogel, were chitosan alone gels presented a less homogenous and more porous structure (Fig. 5). This may be attributed to the drying process, during which the migration of water from the core to the periphery of the gel might have dragged the chitosan to condense on the edge leaving a porous structure in the core. This phenomenon is much less noticeable in the gels containing CNTs, confirming how CNTs have an important role in the formation and preservation of the 3D gel structure. As a consequence, a marked difference in the distribution of the salts deposition was observed and resulted in a much more homogeneous distribution in gels containing CNTs. Quantitative analysis (Fig. 4b) also revealed a higher deposition of salts in CNTs containing gels, with no significant difference between SWNTs and MWNTs (34.6 ± 2.6 and 36.0 ± 1.1 % salt deposition over total sample volume, respectively) [29]. These data are in good agreement with the EDS/SEM data and confirm that calcification is notably more pronounced in samples containing HACS and CNTs.

### Release of model drugs

The release of two macromolecular model drugs, albumin form chicken egg (OVA, Mw 45 kDa, Fig. 4c) and bovine serum albumin (BSA, Mw 66 kDa, Fig. 4d), were studied for 14 days. All the hydrogels tested showed the potential to provide controlled release within the 2 weeks of the experiment. Analysis of the release during the first 5 h revealed very similar release profiles for all gels, indicating that the initial release is governed by the behavior of the chitosan component of the gel. The addition of HACS and CNTs decreased the amount of BSA release over 14 days, but this did not have a significant effect on the release of OVA. This suggests that the mechanism of release for OVA is mainly dependent on the interaction of the protein with the chitosan network. Also gel degradation has an effect, indeed a marked change in the gel structure was noticed after 14 days of BSA release study. Hydrogels before the release study presented a typical porous structure, with well-defined pores of different size; while hydrogels after the release presented a scaly appearance.

## Discussion

Composite thermosensitive chitosan hydrogels were formulated. The addition of CNTs was confirmed to have a significant effect on the sol–gel transition time; these results are in accordance to those obtained by addition of chitosan grafted CNTs [12], indicating that both covalently and non-covalently coated CNTs present similar thermal behaviour and lead to similar effects on the gelation time of the formulations [30, 31]. Unexpectedly, the formulations containing NOSC suspensions of CNTs had better syringeability performance than chitosan only or chitosan with HACS formulation, while previous studies showed that the addition of chitosan grafted CNTs increased the force needed to expel the suspension from the syringe [12]. These data show that covalent and non-covalent coating of CNTs can have very different effect on the viscosity of the suspensions and their syringeability. In our previous work, we demonstrated that NOSC tends to stretch and align along the surface of CNTs [32], reducing the opportunity for formation of physical entanglements between NOSC and the free chitosan chains present in the gel. It is postulated that under the shear stress applied during injection non-covalently coated CNTs are able to align within the flowing liquid formulation therefore present a reduced resistance compared to a crosslinked system. Conversely, CNTs afforded a significant increase in resistance to compression for the composite hydrogels. In comparison with published data, where chitosan grafted CNTs were added instead of non-covalently coated ones, no statistical difference was noticed in the hydrogels containing MWNTs, while SWNTs produced statistically stronger gels (*P* < 0.05) when suspended in NOSC solutions [12]. The use of NOSC for the stabilisation of CNTs reduced the hydrophobicity of the nanotubes when compared to chitosan grafted CNTs. This phenomenon can be explained by considering the shielding effect that NOSC has on the surface of the nanotubes. In fact, five chains of NOSC arrange themselves along the entire surface of CNTs, thus resulting in a net decrease of surface hydrophobicity [21]. Reduced hydrophobicity has been linked with better in vivo outcomes [26, 33, 34]; furthermore, previous studies demonstrated the cytocompatibility of CNTs stabilised in NOSC (data not shown). The in vitro calcification studies revealed that CNTs played a major role in the spatial arrangements of newly formed calcium deposits in the composite materials studied, suggesting that they may have a role in the way the repair of fragile and/or fractured bones occurs in vivo. Finally, in model drug delivery studies, the composite gels demonstrated potential as delivery platforms for macromolecular drugs with minimum interference by CNTs in the determination of the mechanism of drug release.

In conclusion the present study reports of a simple non-covalent method for the inclusion of CNTs into hydrophilic chitosan hydrogels. The non-covalent stabilisation of the nanotubes is advantageous as it facilitates the injectability of the formulation while affording gels with higher compressibility. CNTs also proved to be key in guaranteeing a homogeneous deposition of salts suggesting that they can be used for guided mineralisation of scaffold for bone regeneration. Finally the gels have potential also as platforms for prolonged drug delivery.

## References

[1] Barrère F, Mahmood TA, de Groot K, van Blitterswijk CA (2008). Advanced biomaterials for skeletal tissue regeneration: instructive and smart functions. Mater Sci Eng R.

[2] Lu XY, Qiu T, Wang XF, Zhang M, Gao XL, Li RX (2012). Preparation of foam-like carbon nanotubes/hydroxyapatite composite scaffolds with superparamagnetic properties. Appl Surf Sci.

[3] Porter JR, Ruckh TT, Popat KC (2009). Bone tissue engineering: a review in bone biomimetics and drug delivery strategies. Biotechnol Prog.

[4] Bose S, Roy M, Bandyopadhyay A (2012). Recent advances in bone tissue engineering scaffolds. Trends Biotechnol.

[5] Amini AR, Laurencin CT, Nukavarapu SP (2012). Bone tissue engineering: recent advances and challenges. Crit Rev Biomed Eng.

[6] Klouda L, Mikos AG (2008). Thermoresponsive hydrogels in biomedical applications—a review. Eur j pharm biopharm.

[7] Zhu J, Marchant RE (2011). Design properties of hydrogel tissue-engineering scaffolds. Expert Rev Med Devices.

[8] Im O, Li J, Wang M, Zhang LG, Keidar M (2012). Biomimetic three-dimensional nanocrystalline hydroxyapatite and magnetically synthesized single-walled carbon nanotube chitosan nanocomposite for bone regeneration. Int J Nanomed.

[9] Zhang L, Hu J, Athanasiou KA (2009). The Role of tissue engineering in articular cartilage repair and regeneration. Crit Rev Biomed Eng.

[10] Croisier F, Jérôme C (2013). Chitosan-based biomaterials for tissue engineering. Eur Polym J.

[11] Roldo M, Fatouros GD, Zilberman M (2011). Chitosan derivative based hydrogels as drug delivery platforms. Biomaterials and nanostructures for active implants. studies of mechanobiology, tissue engineering and biomaterials.

[12] Yasmeen S, Lo MK, Bajracharya S, Roldo M (2014). Injectable scaffolds for bone regeneration. Langmuir.

[13] Wang T, Yang X, Qi X, Jiang C (2015). Osteoinduction and proliferation of bone-marrow stromal cells in three-dimensional poly (ε-caprolactone)/hydroxyapatite/collagen scaffolds. J Transl Med.

[14] Verron E, Khairoun I, Guicheux J, Bouler J-M (2010). Calcium phosphate biomaterials as bone drug delivery systems: a review. Drug Disc Today.

[15] Roldo M, Fatouros DG (2013). Biomedical applications of carbon nanotubes. Ann Rep Sec C.

[16] Zancanela DC, de Faria AN, Simao AM, Goncalves RR, Ramos AP, Ciancaglini P (2016). Multi and single walled carbon nanotubes: effects on cell responses and biomineralization of osteoblasts cultures. J Mater Sci Mater Med.

[17] Bianco A, Kostarelos K, Partidos CD, Prato M (2005). Biomedical applications of functionalised carbon nanotubes. Chem Commun.

[18] Shi X, Hudson JL, Spicer PP, Tour JM, Krishnamoorti R, Mikos AG (2006). Injectable nanocomposites of single-walled carbon nanotubes and biodegradable polymers for bone tissue engineering. Biomacromolecules.

[19] Barabás R, Katona G, Bogya ES, Diudea MV, Szentes A, Zsirka B (2015). Preparation and characterization of carboxyl functionalized multiwall carbon nanotubes–hydroxyapatite composites. Ceramics International.

[20] Fatouros GD, Roldo M, Van der Merwe SM, Pastorin G (2011). Stabilisation of carbon nanotube suspensions. Carbon nanotubes: from bench chemistry to promising biomedical applications.

[21] Roldo M, Power K, Smith JR, Cox PA, Papagelis K, Bouropoulos N (2009). N-Octyl-O-sulfate chitosan stabilises single wall carbon nanotubes in aqueous media and bestows biocompatibility. Nanoscale..

[22] Nazar H, Fatouros DG, van der Merwe SM, Bouropoulos N, Avgouropoulos G, Tsibouklis J (2011). Thermosensitive hydrogels for nasal drug delivery: the formulation and characterisation of systems based on N-trimethyl chitosan chloride. Eur J Pharm Biopharm.

[23] Kokubo T, Takadama H (2006). How useful is SBF in predicting in vivo bone bioactivity?. Biomaterials.

[24] Burckbuchler V, Mekhloufi G, Giteau AP, Grossiord JL, Huille S, Agnely F (2010). Rheological and syringeability properties of highly concentrated human polyclonal immunoglobulin solutions. Eur J Pharm Biopharm.

[25] Cilurzo F, Selmin F, Minghetti P, Adami M, Bertoni E, Lauria S (2011). Injectability evaluation: an open issue. AAPS Pharmscitech..

[26] Ormsby R, McNally T, O’Hare P, Burke G, Mitchell C, Dunne N (2012). Fatigue and biocompatibility properties of a poly(methyl methacrylate) bone cement with multi-walled carbon nanotubes. Acta Biomater.

[27] Gierszewska-Drużyńska M, Ostrowska-Czubenko J (2012). Mechanism of water diffusion into noncrosslinked and ionically crosslinked chitosan membranes. Prog Chem Appl Chitin Deriv.

[28] Chavan PN, Bahir MM, Mene RU, Mahabole MP, Khairnar RS (2010). Study of nanobiomaterial hydroxyapatite in simulated body fluid: formation and growth of apatite. Mater Sci Eng B.

[29] Shin SR, Bae H, Cha JM, Mun JY, Chen Y-C, Tekin H (2012). CNT reinforced hybrid microgels as scaffold materials for cell encapsulation. ACS Nano.

[30] Du F, Fischer JE, Winey KI (2003). Coagulation method for preparing single-walled carbon nanotube/poly(methyl methacrylate) composites and their modulus, electrical conductivity, and thermal stability. J Polym Sci B.

[31] Hone J, Llaguno MC, Biercuk MJ, Johnson AT, Batlogg B, Benes Z (2002). Thermal properties of carbon nanotubes and nanotube-based materials. Appl Phys A.

[32] Piovesan S, Cox PA, Smith JR, Fatouros DG, Roldo M (2010). Novel biocompatible chitosan decorated single-walled carbon nanotubes (SWNTs) for biomedical applications: theoretical and experimental investigations. Phy Chem Chem Phys..

[33] Ciapetti G, Granchi D, Devescovi V, Baglio SR, Leonardi E, Martini D (2012). Enhancing osteoconduction of PLLA-based nanocomposite scaffolds for bone regeneration using different biomimetic signals to MSCs. Int J Molecular Sci..

[34] Pan L, Pei X, He R, Wan Q, Wang J (2012). Multiwall carbon nanotubes/polycaprolactone composites for bone tissue engineering application. Colloids Surf B.

